# Transfer of Dicamba Tolerance from *Sinapis arvensis* to *Brassica napus* via Embryo Rescue and Recurrent Backcross Breeding

**DOI:** 10.1371/journal.pone.0141418

**Published:** 2015-11-04

**Authors:** M. Jugulam, Asma Ziauddin, Kenny K. Y. So, Shu Chen, J. Christopher Hall

**Affiliations:** 1 Department of Agronomy, Kansas State University, Manhattan, Kansas, United States of America; 2 School of Environmental Sciences, University of Guelph, Guelph, Ontario, Canada; 3 Laboratory Services Division, University of Guelph, Guelph, Ontario, Canada; Chungnam National University, REPUBLIC OF KOREA

## Abstract

Auxinic herbicides (*e*.*g*. dicamba) are extensively used in agriculture to selectively control broadleaf weeds. Although cultivated species of Brassicaceae (e.g. Canola) are susceptible to auxinic herbicides, some biotypes of *Sinapis arvensis* (wild mustard) were found dicamba resistant in Canada. In this research, dicamba tolerance from wild mustard was introgressed into canola through embryo rescue followed by conventional breeding. Intergeneric hybrids between *S*. *arvensis* (2n = 18) and *B*. *napus* (2n = 38) were produced through embryo rescue. Embryo formation and hybrid plant regeneration was achieved. Transfer of dicamba tolerance from *S*. *arvensis* into the hybrid plants was determined by molecular analysis and at the whole plant level. Dicamba tolerance was introgressed into *B*. *napus* by backcrossing for seven generations. Homozygous dicamba-tolerant *B*. *napus* lines were identified. The ploidy of the hybrid progeny was assessed by flow cytometry. Finally, introgression of the piece of DNA possibly containing the dicamba tolerance gene into *B*. *napus* was confirmed using florescence in situ hybridization (FISH). This research demonstrates for the first time stable introgression of dicamba tolerance from *S*. *arvensis* into *B*. *napus* via *in vitro* embryo rescue followed by repeated backcross breeding. Creation of dicamba-tolerant *B*. *napus* varieties by this approach may have potential to provide options to growers to choose a desirable herbicide-tolerant technology. Furthermore, adoption of such technology facilitates effective weed control, less tillage, and possibly minimize evolution of herbicide resistant weeds.

## Introduction

Auxinic herbicides were the first synthetic herbicides discovered and have been in use for several decades in agriculture. These herbicides have been a favorite choice among farmers worldwide as they selectively control broadleaf weeds. As such, auxinic herbicides cannot be used in broadleaf crops. Despite seven decades of extensive use, only 32 auxinic herbicide-resistant biotypes have been documented worldwide [1]; a relatively low number compared to herbicides with other modes of action such as acetolactate synthase (ALS)-inhibitors or triazines. Due to intense selection pressure, *Sinapis arvensis* (wild mustard) biotypes evolved resistance to auxinic herbicides in western Canadian cereal fields [2]. These auxinic herbicide-resistant *S*. *arvensis* biotypes were found to be highly resistant to both picloram and dicamba (104 fold) [2]. Following extensive morphological, physiological, biochemical, and molecular characterization, these *S*. *arvensis* auxinic herbicide-resistant biotypes have been used as a model species to study the mechanism of resistance and the mode of action of auxinic herbicides [3–5]. Genetic analysis of auxinic herbicide-resistant *S*. *arvensis* suggests that the resistance trait behaves as a single dominant Mendelian gene [6,7]. Furthermore, we recently reported the identification of morphological and molecular markers-linked to the dicamba resistance trait in *S*. *arvensis* [8].

Brassica crops are among the oldest crop species in agronomic history, with records of their cultivation dating back to 1500 BC [9]. Among the approximately 350 genera within Brassicaceae are numerous economically important oil and vegetable crops. Canola, a *Brassica napus* variety developed for its low eurcic acid, is one of the world’s most important oilseed crops [10]. Seed yield in *B*. *napus* crops is highly sensitive to weed competition, and poor weed management results in significant reduction in yield [11]. The successful introduction and adoption of glyphosate- and glyphosate-resistant canola varieties reflects the canola industry’s demand and need for simple and effective weed management. Despite its initial success, the long term viability of cropping glyphosate-resistant canola has come under scrutiny as reports of glyphosate-resistant weeds have become increasingly prevalent in the last decade [1]. One of the primary reasons for the development of glyphosate resistance in weeds is due to the sole and continual use of glyphosate in glyphosate–resistant cropping systems [12]. The continuous use of herbicides with the same mode of action creates selection pressure and results in the evolution of herbicide-resistant biotypes in weeds naturally found in these crop fields. The availability of cultivars resistant to herbicides with different mechanisms of action will allow farmers to rotate herbicides between crops thereby breaking selection pressure and effectively minimizing the possibility of evolving herbicide resistance in nearby weed species.

Dicamba is an important auxinic herbicide in monocotyledonous cropping systems such as corn and other cereals. Dicamba is used both as a pre-emergence and post emergence herbicide for selectively controlling broadleaf weeds. During the past several decades, the demand for dicamba in agriculture has remained consistent despite the introduction of herbicides (glyphosate, triazines, and ALS-inhibitors) with different modes of action primarily because of its selectivity, efficacy, and low application costs [13]. Selectivity for broadleaf weeds is also a major limitation in the use of dicamba as it cannot be used in broadleaf crops such as canola. The availability of dicamba-resistant canola cultivars will not only offer canola farmers an alternative low-cost herbicide option for crop protection, but may reduce the evolution of weeds resistant to current herbicides.

A number of intergeneric hybrids were developed previously between *S*. *arvensis* and *B*. *napus* [14–16]. Hu et al. [17] reported that intergeneric hybrids between *B*. *napus* and *S*. *arvensis* could be produced by using protoplast fusion. The numbers of hybrid progeny reported in these studies were low and most of the progeny were sterile. The overall goal of our research was to transfer dicamba tolerance from *S*. *arvensis* to *B*. *napus* by means of conventional breeding. The specific objectives were to: a) produce intergeneric hybrids between dicamba-resistant *S*. *arvensis* and *B*. *napus* via embryo rescue b) introgress dicamba tolerance into *B*. *napus* through backcrossing and c) demonstrate the stable introgression of dicamba tolerance into *B*. *napus*.

## Methods

### Production of hybrids from auxinic-herbicide resistant *S*. *arvensis* and *B*. *napus* crosses

Dicamba-resistant *S*. *arvensis* and -susceptible *B*. *napus* were raised from seed in six inch plastic pots containing Promix (Plant Products, Brampton, Canada). All plants were grown in a growth chamber under a 16-h photoperiod with a light intensity of 350 μmoles.s^-1^m^-2^ and 22/15°C day/night temperatures. Plants were irrigated as required and fertilized weekly with 20-8-20 fertilizer. To confirm dicamba resistance, plants of *S*. *arvensis* were treated by spraying with dicamba (Banvel, BASF, USA) at 200 g acid equivalent (ae)/ha at the three- to four-leaf stage (procedure for dicamba application below).

Intergeneric F_1_ hybrids between dicamba-resistant *S*. *arvensis* and *B*. *napus* were produced through reciprocal crosses following the procedures described by Jugulam et al. [7]. Embryo rescue was required as hybrid seeds failed to develop. To optimize hybrid plant regeneration, two embryo rescue protocols, i.e., embryo culture and silique culture, were used according to Mithila and Hall [18]. Established plantlets were transferred to soil and were grown in the growth chamber.

### Assessment of transfer of dicamba tolerance into hybrids

#### Molecular assay

We developed a genetic map describing AFLP (amplified fragment length polymorphism) molecular markers closely-linked to dicamba tolerance in *S*. *arvensis* [8]. The two closest markers that were identified and sequenced were 1.58 and—6.35 map units flanking the resistance locus, and were designated M5 and M2, respectively [8]. To confirm the presence of marker closely-linked to dicamba tolerance in the hybrid progeny, PCR was conducted using primers generated from the M5 molecular marker (forward primer: *GGCCGCGAGACATTGGTGA* and reverse primer: *TCTCTCGTGACCCTTTACAATTAG* *)*. The expected amplicon size was 225 bp. Genomic DNA was extracted from the leaf tissue of hybrid progeny and their parental plants using DNeasy^®^ Plant Mini (QIAGEN, Mississauga, ON, Canada). Thirty cycles of PCR were conducted using the following conditions: initial denaturation at 94°C for 3 min, followed by 30 cycles of 94°C for 30 seconds, 56°C for 30 sec and 72°C for 45 sec with a final extension at 72°C for 7 min. Dicamba tolerance was determined by the presence of the expected 225 bp amplification product.

#### Whole-plant assay

Dicamba-resistant *S*. *arvensis*, susceptible *B*. *napus* and hybrid plants were grown and treated with dicamba 200 g ae ha^-1^ at the three- to four- leaf stage using a motorized hood sprayer. The sprayer was equipped with a flat-fan nozzle (8002 E) and calibrated to a spray rate of 200 l/ha. One and two weeks post-spray, seedlings were visually rated for injury, indicated by foliar epinasty (downward curlingof plant parts and a typical symptom of auxinic herbicides) and senescence. Hybrid plants were classified dicamba-tolerant or -susceptible by comparing the injury response with those of dicamba-resistant *S*. *arvensis* and–susceptible *B*. *napus* plants.

### Assessment of fertility of hybrids

Pollen fertility/viability and stigma receptivity of dicamba-tolerant hybrids were assessed. Acetocarmine stain was used to test pollen viability in the hybrid progeny. Upon anthesis, the flowers were collected and the pollen was transferred onto a microscope slide, stained with 1% acetocarmine, and observed under a compound microscope (40X). Stigma receptivity of the hybrid plants was tested by performing crosses with wild-type *B*. *napus* as the pollen donor.

### Repeated backcrosses to introgress dicamba tolerance from hybrids into *B*. *napus*

Backcrosses were performed using the dicamba-tolerant intergeneric hybrid as the donor parent and *B*. *napus* as the recurrent parent. BC_3_F_1_ plants were grown and treated with three doses of dicamba; i.e. 200, 400 or 600 g ae ha^-1^ at the three- to four- leaf stage (as described above). However, progeny from all seven backcross generation were tested with 200 g ae ha^-1^ dicamba. Chi square analysis was performed by SAS (version 9.3; Cary, NC) to determine the goodness of fit to a 1:1 segregation, i.e. tolerance: susceptible. After seven backcrosses, homozygous dicamba-tolerant *B*. *napus* plants were identified through three subsequent generations of self-pollination. Dicamba tolerance was confirmed via whole plant screening with dicamba spray and PCR analysis of the tolerant plants.

### DNA ploidy of hybrids and backcross progeny

The DNA ploidy of the initial intergeneric hybrids and their backcrossed progeny were assessed by flow cytometry following the protocol of Kron et al. [19]. Dicamba-resistant *S*. *arvensis* (diploid) and *B*. *napus* (tetraploid) served as controls.

### Florescent in situ hybridization

Fluorescent in situ hybridization (FISH) was performed to confirm the integration of the dicamba tolerance gene into the nuclear genome of the dicamba-tolerant *B*. *napus* plants. Homozygous dicamba-tolerant *B*. *napus* line (11-12-8) seeds were germinated on wet filter paper at room temperature in the dark for 2–3 days. Root-tips (5–7 mm) were harvested and fixed in Carnoy’s I fixative (3 parts 95% ethanol: 1 part acetic acid) for a minimum of one hour and no longer than sixteen hours. For chromosome preparation, the meristematic area of the root tip was excised and macerated in 45% acetic acid on a glass slide, then subsequently mounted with a glass coverslip. The eraser end of a wooden pencil was used to tap and dispersed the root tissue evenly. Slides were observed under a phase contrast microscope and those containing cells with full chromosome complements were stored at -80°C.

The fluorescent probe, produced from the 225 bp PCR product previously amplified, was labeled with Cy3 fluorochrome dCTP. Coverslips were removed from the frozen slide with a razor blade, and slides were placed in a 70% and 95% ethanol series to dehydrate the tissue, then air dried. RNA contamination was removed by incubating slides at 37°C for 60 min with RNase solution diluted to 100 μg/ml in 2X saline-sodium citrate (SSC) buffer. Tissues on slides were washed in 2X SSC, dehydrated in the ethanol series (70% and 95%), and air dried. The hybridization mixture was prepared with 50% de-ionized formamide, 10% dextran sulfate, 2X SSC and probe DNA. The hybridization solution was denatured at 75°C for 10 min and cooled on ice prior to being applied to chromosome preparations at a concentration of 100 ng of probe DNA per slide. The coverslips on each slide was sealed with liquid cement and incubated for 10 min at 80°C to denature chromosomal DNA. Hybridization occurred over 16 hours at 37°C in a sealed humidity chamber. Subsequently, slide covers were removed and slides were washed for 10 min in 2X SSC at room temperature, followed by two 15- min washes in 2X SSC at 37°C, concluding with a final wash in 4X SSC plus 0.2% Tween 20 surfactant for 10 min before air drying. The slides were mounted with Vectashield mounting media (Vector Labs) containing 4, 6-diamino-2-phenylindole (DAPI). DAPI (excitation at 360 nm and emission at 460 nm) fluoresces blue when bound to DNA. Cy3 fluorochrome (excitation at 550 nm and emission at 570 nm) fluoresces orange/red. The fluorescence signals were visualized using a Leica DM 5500 fluorescent microscope.

## Results

### Production of hybrids between *S*. *arvensis* and *B*. *napus*

**Fig 1:**
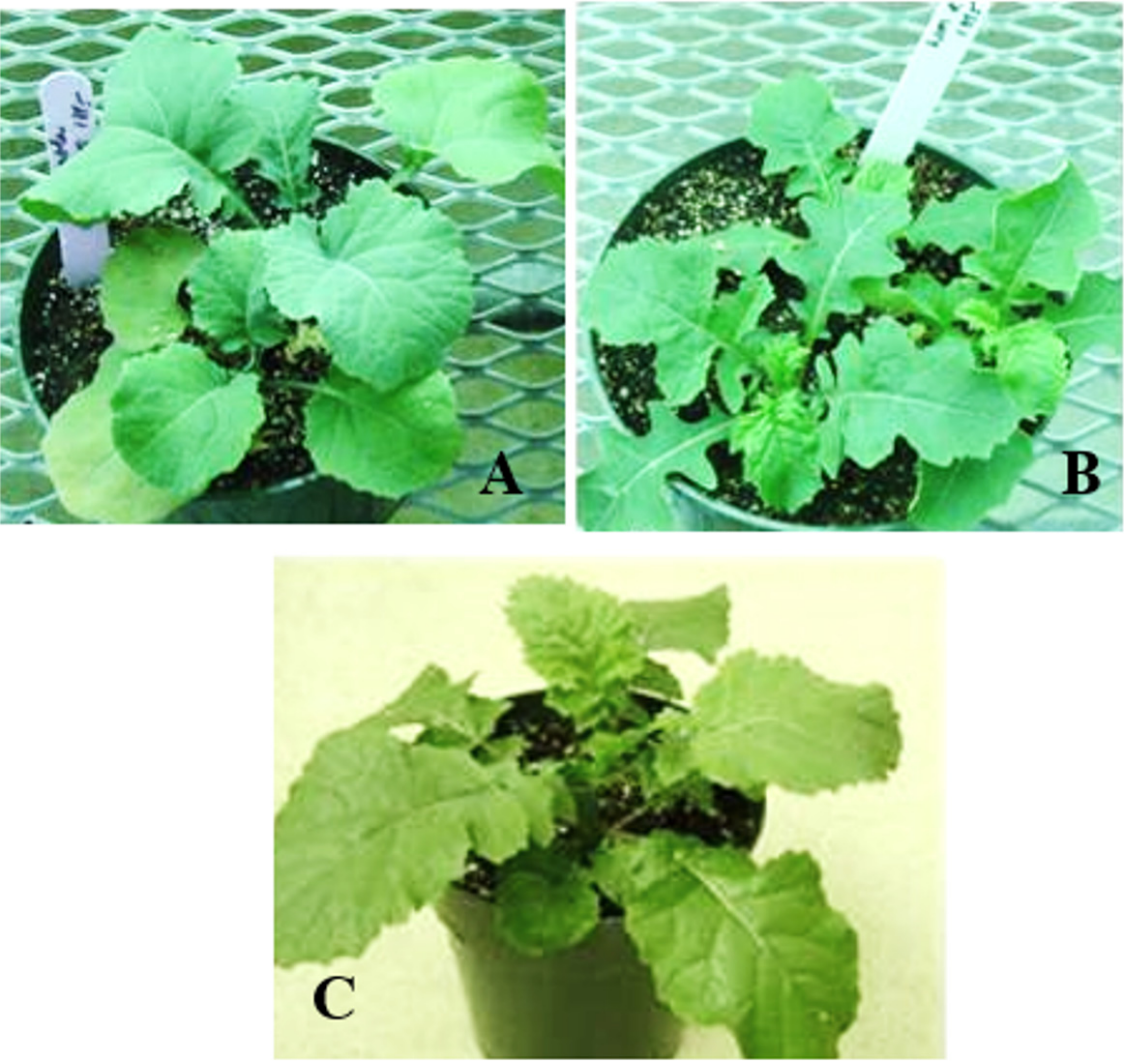
Production of hybrids between *B*. *napus* and *S*. *arvensis*. A and B represent *B*. *napus* and *S*. *arvensis* plants, respectively. C is a triploid hybrid plant produced by crossing *B*. *napus* and *S*. *arvensis*.

Among two embryo rescue protocols used as described by Mithila and Hall [18], strategy II (i.e. culturing of immature siliques on medium prior to embryo rescue) was best for producing *B*. *napus* x *S*. *arvensis* hybrids (Table 1). Hybrids were produced only when *B*. *napus* was used as the maternal parent. The progeny exhibited several morphological traits of both the parents e.g. leaf shape, stem, plant height and flower color (Fig 1).

**Table 1:** Hybrid production between *Brassica napus* and *Sinapis arvensis*: frequency of embryo regeneration and hybrid plant establishment upon direct and reciprocal crosses followed by *in vitro* culture and embryo rescue.

Cross Combination	# of buds pollinated	# of siliques cultured	# of embryos excised	# of embryos germinated	# of hybrids obtained
***Strategy I***					
*1*. *B*. *napus* X *S*. *arvensis*	200	-	60	2	2
*2*. *S*. *arvensis* X *B*. *napus*	200	-	30	0	0
***Strategy II***					
*1*. *B*. *napus* X *S*. *arvensis*	150	110	55	9	9
*2*. *S*. *arvensis* X *B*. *napus*	150	120	10	0	0

Strategy I: Siliques harvested 10–12 days after pollination, embryos excised and cultured on media. Strategy II: Immature siliques harvested 3–5 days after pollination, cultured on media for 2 weeks and after which embryos were harvested and cultured on media.

### Assessment of dicamba tolerance in hybrids

PCR results demonstrated successful amplification of the expected 225 bp fragment, closely-linked to dicamba tolerance in *S*. *arvensis* and hybrids (S1 Fig). Sixty-seven percent of the hybrid plants were found to contain the marker closely-linked to dicamba tolerance and also survived dicamba (200 g ae ha^-1^) application.

### Assessment of fertility and DNA ploidy of hybrids and backcross progeny

**Fig 2:**
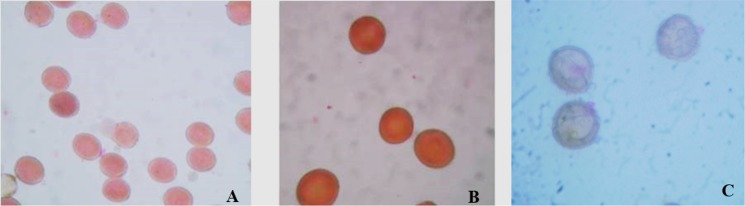
Pollen viability test (stained with 1% acetocarmine). A and B are viable pollen of *B*. *napus* and hybrid, respectively; whereas, C represents non-viable hybrid pollen. Pictures were taken at 40X magnification.

Pollen/stigma fertility tests demonstrated that among the hybrids that were found tolerant to dicamba, only 20% and 50% were male and female fertile, respectively. When stained with acetocarmine, viable pollen appeared stained and spherical in shape; conversely, non-viable pollen was colorless (Fig 2). The male fertile hybrids were maintained *in vitro* by clonal propagation. Flow cytometry data indicated the the F_1_ hybrids were all triploids with an estimated ploidy range of 3.1 to 3.2 (triploid); while the parents *S*. *arvensis* and *B*. *napus* had an estimated ploidy of 1.88 to 1.9, (diploid) and 4 to 4.01 (tetraploid) (S1 Table), respectively. However, the progeny from the initial few backcrosses consisted of either tetraploids, pentaploids, or anueploids and a large number of plants from backcross four onwards were found to be tetraploids (S1 Table).

### Assessment of dicamba tolerance and segregation in backcrosses progeny

**Fig 3:**
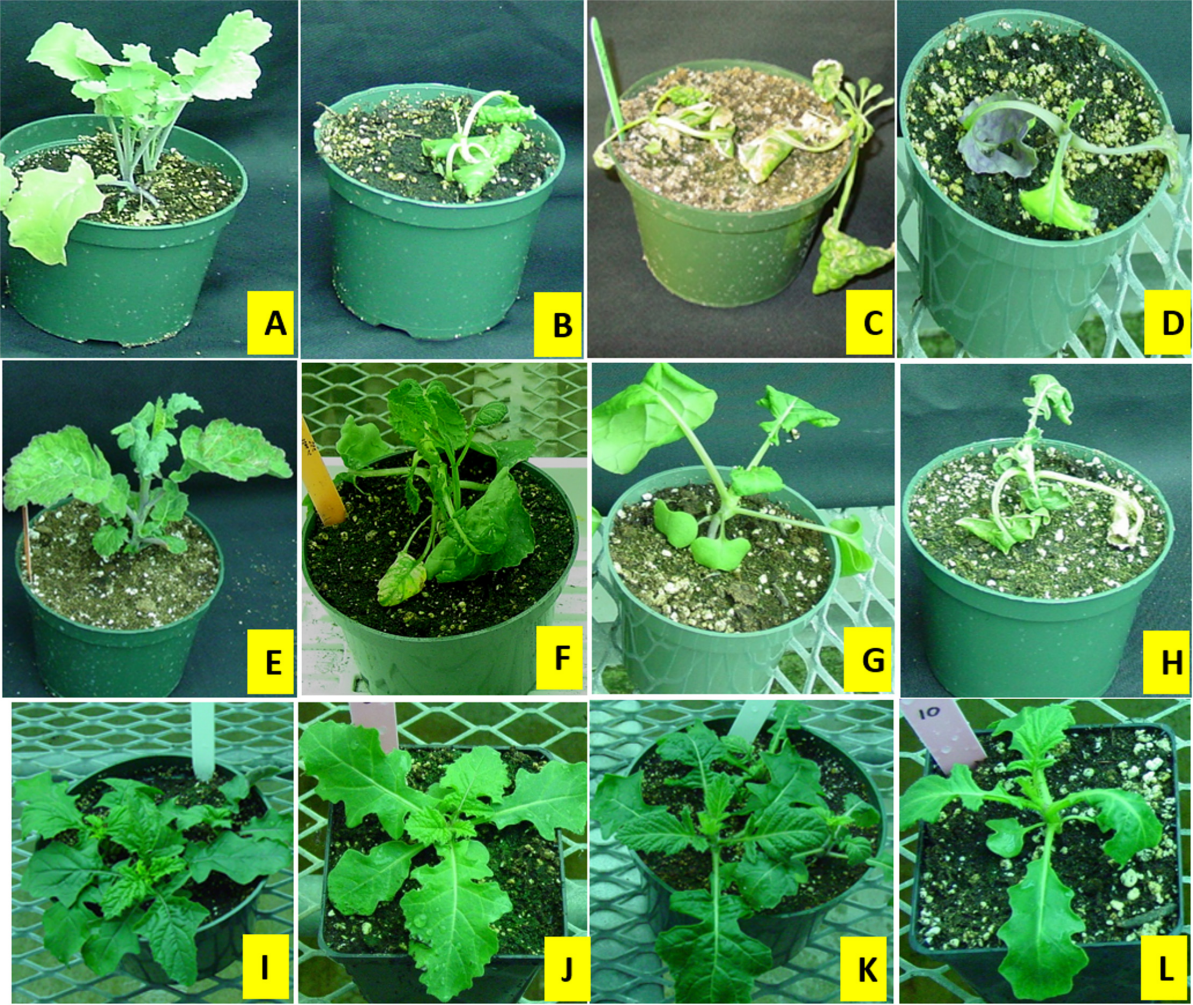
Plant response to dicamba (200, 400 or 600 g ae ha^-1^) 2 weeks after treatment. Top and bottom row represent *B*. *napus* and *S*. *arvensis*, respectively. The middle row shows the response of dicamba-tolerant BC_3_F_1_ hybrids. A, E and I are untreated plants; B, F and J illustrate plant response to 200 g ae ha^-1^; C, G and K represent plant response to 400 g ae ha^-1^; whereas, D, H, L indicate plant response to 600 g ae ha^-1^.

**Fig 4:**
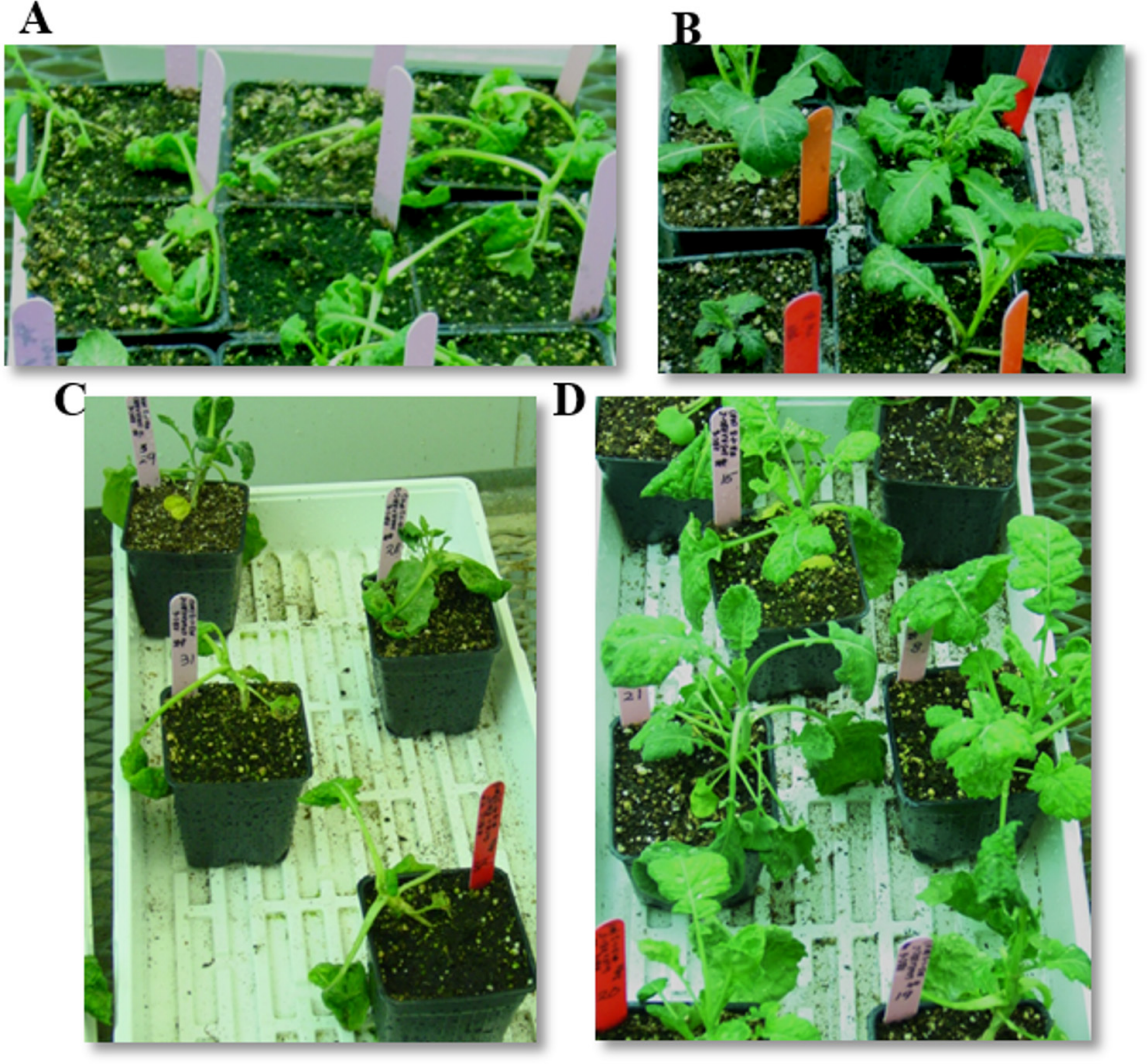
Plant response to dicamba (200 g ae ha^-1^) 2 weeks after treatment. A, B, represent *B*. *napus* (epinasty) and *S*. *arvensis* (no epinasty), respectively. C and D segregation of dicamba-susceptible (epinasty) and–tolerant (no epinasty) plants in BC_6_F_1_ generation, respectively.

The dose-response of BC_3_F_1_ hybrids suggests that some of the hybrid plants were tolerant to dicamba up to 400 g ae ha^-1^ with their response comparable to dicamba-resistant *S*. *arvensis* treated with the same dose (Fig 3) and others were severely injured and eventually, died. However, the tolerant hybrids were found slightly injured at 600 g ae ha^-1^ dicamba (Fig 3). The progeny from other backcrosses showed varying responses following 200 g ae ha^-1^ dicamba application. Some seedlings exhibited severe epinasty one week post spray and eventually died while others showed only minor dicamba injury initially and recovered. When progeny from each backcross were treated with dicamba, based on the probabilities, plants in backcrosses 5, 6 and 7 appear to segregate 1:1 for R: S (Table 2 and Fig 4) as expected for a single dominant Mendelian trait.

**Table 2:** Response of backcross progeny upon dicamba treatment and evaluation of fertility of the progeny. Segregation of resistant (R) and susceptible (S) plants in backcross progeny following treatment with 200 g ae/ha of dicamba.

*Backcross*	*Segregation of plants*		*χ* ^*2*^	*P-value*	*Fertility of R plants*
	*R*	*S*			
BC_1_F_1_	5	0	-	-	Plants were pollen fertile and female sterile
BC_2_F_1_	4	5	0.11	0.7389	Only 1 R plant was pollen fertile
BC_3_F_1_	30	9	11.3	0.0008	17 R plants were pollen fertile
BC_4_F_1_	9	26	8.25	0.0041	All R plants were pollen fertile
BC_5_F_1_	19	15	0.47	0.4927	All R plants were pollen fertile
BC_6_F_1_	17	13	0.53	0.4652	All R plants were pollen fertile
BC_7_F_1_	17	16	0.03	0.8618	All R plants were pollen fertile

***χ*** ^***2***^:Chi-square values are the results of tests for goodness of fit to a 1:1 (R:S) segregation model. **P**-value significance is determined at < 0.05.

### Identifying homozygous dicamba-tolerant *B*. *napus* lines

A homozygous dicamba-tolerant BC_7_F_3_ line (11-12-8) was identified when all the progeny tested from this line were tolerant to dicamba spray (200 g ae ha^-1^). Furthermore, PCR analysis of all the sprayed plants in line 11-12-8 showed the presence and amplification of the M5 molecular marker (225 bp; S2 Fig). The estimated DNA ploidy (based on flow cytometry data) of this line (S1 Table) was similar to that of *B*. *napus* suggesting that the amount of genetic material transferred from *S*. *arvensis* to *B*. *napus* was minimal.

### Fluorescent in situ hybridization

**Fig 5:**
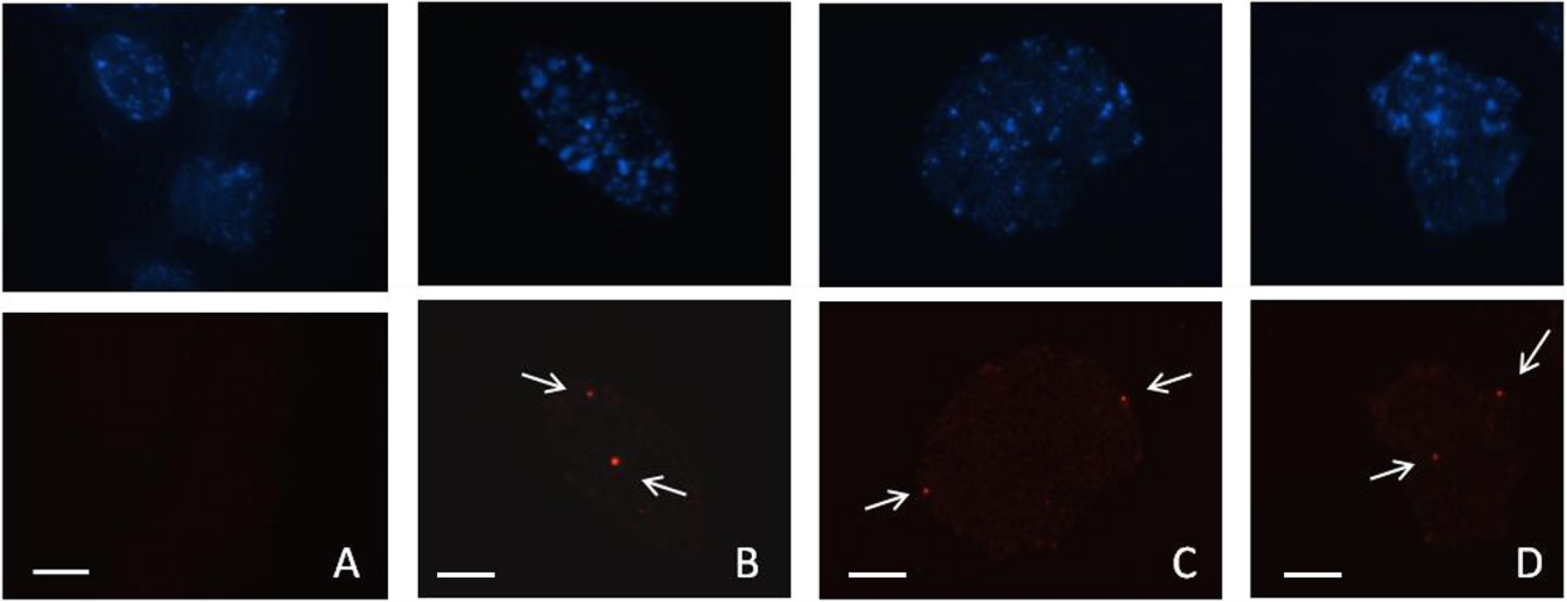
Fluorescence in situ hybridization of a 225 bp introgressed from *S*. *arvensis* labeled with a Cy3 fluorochrome. Panel A is the prophase stage cells from *B*. *napus*. Panels B-D are prophase stage cells from the root-tips of *B*. *napus* BC_7_F_3_ line (11-12-8). The top row in each panel is visualized with a filter for DAPI where DNA fluoresces blue. The bottom panel is the same cell visualized with a filter that detects Cy3 fluorescence; each cell in BC_7_F_3_ line (11-12-8) has two red/orange fluorescing dots (arrows) suggesting a homologous insertion. Bar = 1 μm.

A short probe (225 bp) linked to the M5 molecular marker directly labeled with a Cy3 fluorochrome was detected in the hybridized *B*. *napus* line (11-12-8) chromosome suggesting the dicamba-tolerant gene was integrated into the nuclear genome of *B*. *napus*. Fluoresence of the Cy3 probe, indicated by reddish- orange dots (Fig 5), was clearly visible in individual root cells of the dicamba-tolerant *B*. *napus* line. No fluorescence was observed in the nuclear material of the dicamba-susceptible *B*. *napus* control (Fig 5A). Within each intact cell, two spatially separated red fluorescing points were observed indicating the presence of the transferred gene in a homozygous state on two homologous chromosomes (Fig 5B–5D).

## Discussion

Successful gene transfer from intergeneric and interspecific crosses using conventional plant breeding techniques requires overcoming several barriers, including pollen stigma incompatibility, embryo/endosperm imbalance, sterility, and a lack of homologous chromosome pairing between the species. Cytogenetic analyses of *B*. *napus* and *S*. *arvensis* hybrids by Mizusima [20, 21] suggested that partial homologies can be established on the basis of allosyndetic pairing. In this research we produced fertile intergeneric hybrids between dicamba-susceptible *B*. *napus* and dicamba-resistant *S*. *arvensis* and conducted a series of backcrosses to produce a dicamba-tolerant *B*. *napus* line.

The BC_1_F_1_ plants showed a dose-dependent response to dicamba application (Fig 3), possibly because there may be some other genes from *B*. *napus* influencing the phenotype response upon treatment with dicamba. The observed Mendelian segregation ratios for dicamba tolerance or susceptibility in the later generations of backcrossed progeny (BC_5_F_1_, BC_6_F_1_, BC_7_F_1_) followed expected segregation of a single dominant trait and confirms earlier work of Jasieniuk et al. [6] where dicamba resistance in *S*. *arvensis* was found to be controlled by a single dominant nuclear allele. However, the segregation of dicamba tolerance or susceptibility in initial backcross progeny (i.e. BC_1_F_1_ to BC_4_F_1_), did not fit single gene inheritance configuration (Table 2). This could be because in BC_3_F_1_ there were more aneuploids than tetraploids (S1 Table). Dicamba tolerance was also confirmed by the presence of the M5 molecular marker (which was found closely linked to dicamba resistance in *S*. *arvensis*; Mithila et al. [8]) in all dicamba-tolerant progeny from homozygous BC_7_F_3_ line (11-12-8), suggesting a true-breeding dicamba-tolerant line. These homozygous BC_7_F_3_ plants that survived dicamba application (200 g ae/ha) also completed their life cycle with normal seed production (data not shown). However, future studies are required to assess fitness of these plants in terms of biomass and seed production in comparison with dicamba-susceptible *B*. *napus*.

FISH analysis confirmed the transfer of the wild-mustard DNA segment associated with dicamba tolerance into our *B*. *napus* line. Prophase stage cells from *B*. *napus* root-tips were well suited for retaining the intact chromosome complement and were used routinely in our FISH analysis. Cells at metaphase stage did not give us a well spread full complement of chromosomes most likely as the nuclear envelope is fragmented and the chromosomes tended to be dispersed. We were able to successfully use a very short 225 nucleotide probe directly labeled with Cy3 fluorochrome to detect the transfer of our herbicide resistant gene into the nuclear DNA into our introgressed *B*. *napus* line (Fig 5B–5D). Due to limited seed source and lack of good root-tip preparations, we were unable to perform FISH analysis with the donor resistant wild mustard. However, absence of fluorescent signal in the negative control, dicamba-susceptible canola (Fig 5A), confirms the specificity of the probe to the dicamba-tolerant trait that was introgressed. Unlike indirect labeled probes, the use of a direct labeled probe allowed for faster hybridization as a secondary detection molecule was not required to visualize the fluorescent signal. Furthermore, consistent signal presence and improved signal to background ratios have been reported with the direct method of labeling [22]. In contrast to the majority of relevant literature where FISH has been used to detect large (3–60 kb) or high copy inserts such as ribosomal RNA, our approach of FISH to detect a relatively small insert (0.3 kb) is unique. Large DNA fragments produce stronger signals but often contain repetitive sequences that make it difficult to detect specific target loci [23]. The decision to detect a small insert based off an AFLP marker ensured greater specificity. Furthermore, it has been suggested that a short probe (220–600 bp) allows for better penetration to the DNA packaged within the chromosome and may explain our success [24]. With respect to the use of Cy3 fluorochrome tagging, Cy3 is superior to classical dyes such as Fluorescein-isothiocyanate (FITC) and Tetramethylrhodamine (TRITC) as it has significantly brighter fluorescence and is stable to photobleaching [25]. The combination of a directly labeled short nucleotide probe along with the use of Cy3 fluorochrome enabled the successful visualization of a segment of DNA potentially consisting of dicamba-tolerance that was transferred from *S*. *arvensis* into *B*. *napus*. Furthermore, future studies using high-density single nucleotide polymorphism (SNP) genotyping array [26] can be applied to detect the introgressed DNA segment from *S*. *arvensis* into *B*. *napus*.

As the donor biotype of *S*. *arvensis* was resistant to the field use rate of dicamba (500 g ae/ha), the homozygous dicamba-tolerant line of *B*. *napus* is promising for field evaluation for the performance of the trait introgressed. Further studies evaluating agronomic performance of *B*. *napus* lines possessing dicamba-tolerance will determine the potential use of this technology as a weed management strategy in agriculture. Recently, cost-effective dicamba and 2,4-D-tolerant transgenic crop varieties were developed by multinational agro-based industries. The dicamba-tolerant soybean was developed by introducing a gene coding for an enzyme dicamba O-demethylase from a soil bacterium *Pseudomonas maltophilia* that can rapidly metabolize dicamba [27]. Development of dicamba-tolerant maize with insertion of dicamba O-demethylase gene provided high level of tolerance to dicamba [28]. Furthermore, 2,4-D-tolerant corn was developed by introducing an enzyme aryloxyalkanoate dioxygenase from yet another soil bacterium *Ralstonia eutropha* that can cleave 2,4-D into non-herbicidal form [29].

Although, glyphosate-tolerant *B*. *napus* cultivars have been in use across N. America for the greater part of the last few decades, continuous use of herbicides with a similar mechanism of action creates selection pressure thereby facilitating the evolution of herbicide-resistant weeds. Therefore, the availability of high performing crop cultivars resistant to herbicides of different mechanisms of actions will allow for the rotation of herbicides, a crucial and effective strategy to minimize the development of herbicide-resistant weeds. Furthermore, dicamba-resistant biotype of *S*. *arvensis* used for the generation of our dicamba-tolerant *B*. *napus* is highly resistant to dicamba and picloram but not to mecoprop, another auxinic herbicide [3]. Therefore, if and when there is any occurrence of volunteer dicamba-tolerant *B*. *napus*, mecoprop can still be used to control such volunteers [30].

## Conclusions

In conclusion, this research demonstrated for the first time the transfer of dicamba-tolerant trait from *S*. *arvensis* into a *B*. *napus* line. We have presented a proof of concept breeding scheme to transfer dicamba-tolerant trait from a weed relative into crop species. More importantly, our FISH assay using the combination of a directly labeled short nucleotide probe along with Cy3 fluorochrome facilitated the successful visualization of a segment of DNA potentially consisting of dicamba-tolerance that was transferred from *S*. *arvensis* into *B*. *napus*. Herbicide-resistant agronomic weeds offer valuable source of germplasm for breeding programs. The backcross breeding scheme outlined in this research for introgressing dicamba tolerance is not limited to *B*. *napus* and can be applied to all agricultural and horticultural crops, and their related species. Finally, development and adoption of herbicide-tolerant technology facilitates effective weed control, less tillage, and possibly minimizes evolution of herbicide resistant weeds.

## Supporting Information

S1 Table Estimation of DNA content and ploidy of samples of parents and backcross progeny as determined by flowcytometry (DOCX)

S1 Fig PCR analysis of *B*. *napus* x *S*. *arvensis* hybrids for a 225 bp product corresponding to a marker linked to dicamba resistance in *S*. *arvensis*. Lane 1: 100 bp ladder; 2: reaction without a template; 3: *S*. *arvensis* dicamba-resistant; 4: *B*. *napus*; 6, 7, 9, 10, 11 are hybrids generated via embryo rescue: 6, 10, 11 represent dicamba-tolerant hybrids, whereas, 7, 9 are dicamba-susceptible; 5 and 8: empty wells.
(TIF)

S2 Fig PCR analysis of a BC_7_F_3_ line (11-12-8) for a 225 bp corresponding to a marker linked to dicamba-tolerance in *S*. *arvensis*. Lane 1: 100bp ladder; Lane 2: *S*. *arvensis*- dicamba susceptible; 3: wild-type *B*. *napus*; 4–13: Ten BC_7_F_3_ plants; 14, 15: *S*. *arvensis*-dicamba resistant; 16: reaction without a template.
(TIF)

## Abbreviations

AFLP: amplified fragment length polymorphism
BC: backcross
DAPI: 4, 6-diamino-2-phenylindole
FISH: fluorescent in situ hybridization
FITC: fluorescein-isothiocyanate
SSC: saline-sodium citrate
TRITC: tetramethylrhodamine
