# Supplementary material for: Transfer of Dicamba Tolerance from Sinapis arvensis to Brassica napus via Embryo Rescue and Recurrent Backcross Breeding
Source: PLoS One. 2015 Nov 4;10(11):e0141418. doi: 10.1371/journal.pone.0141418 (PMC4633294; doi:10.1371/journal.pone.0141418)
Supplement: S1 Table — (DOCX) [file pone.0141418.s001.docx]

**Supplemental Information:**

S1 Table:

| **Parents/Backcross Progeny** | **DNA (pg) and Ploidy** |
| --- | --- |
| **Wild Mustard** | 1.13 -1.23 pg; Diploid (2x) |
| **Canola** | 2.38-2.42 pg; Tetraploids (4x) |
| **Hybrid** | 1.69- 1.73 pg; Triploid (3X) |
| **BC_1_F_1_** | 2.30, 2.88, 2.60, 2.89, 2.60 pg ; Tetraploids (4x), Pentaploids (5x) as well as Aneuploids (4.6x, 4.2x, 4.4x, 4.8x, 5.1x etc) |
| **BC_2_F_1_** | 2.70, 2.57, 2.65, 2.58 pg; mostly, aneuploids (4.2x, 4.3x, 4.4x, 4.6x etc) |
| **BC_3_F_1_** | 2.56, 2.49, 2.58, 2.60 pg; mostly, aneuploids (4.2x, 4.3x, 4.4x, 4.7x etc) |
| **BC_4_F_1_** | 2.44, 2.42, 2.48 pg; tetraploids and aneuploids |
| **BC_5_F_1_** | 2.41, 2.40, 2.49 pg; tetraploids and some aneuploids |
| **BC_6_F_1_** | 2.44, 2.45, 2.39, 2.47 pg; mostly tetraploids |
| **BC_7_F_1_** | 2.42, 2.44, 2.39, 2.43 pg; all are tetraploids |
