# Supplementary figures and images for: Transfer of Dicamba Tolerance from Sinapis arvensis to Brassica napus via Embryo Rescue and Recurrent Backcross Breeding
Source: PLoS One. 2015 Nov 4;10(11):e0141418. doi: 10.1371/journal.pone.0141418 (PMC4633294; doi:10.1371/journal.pone.0141418)

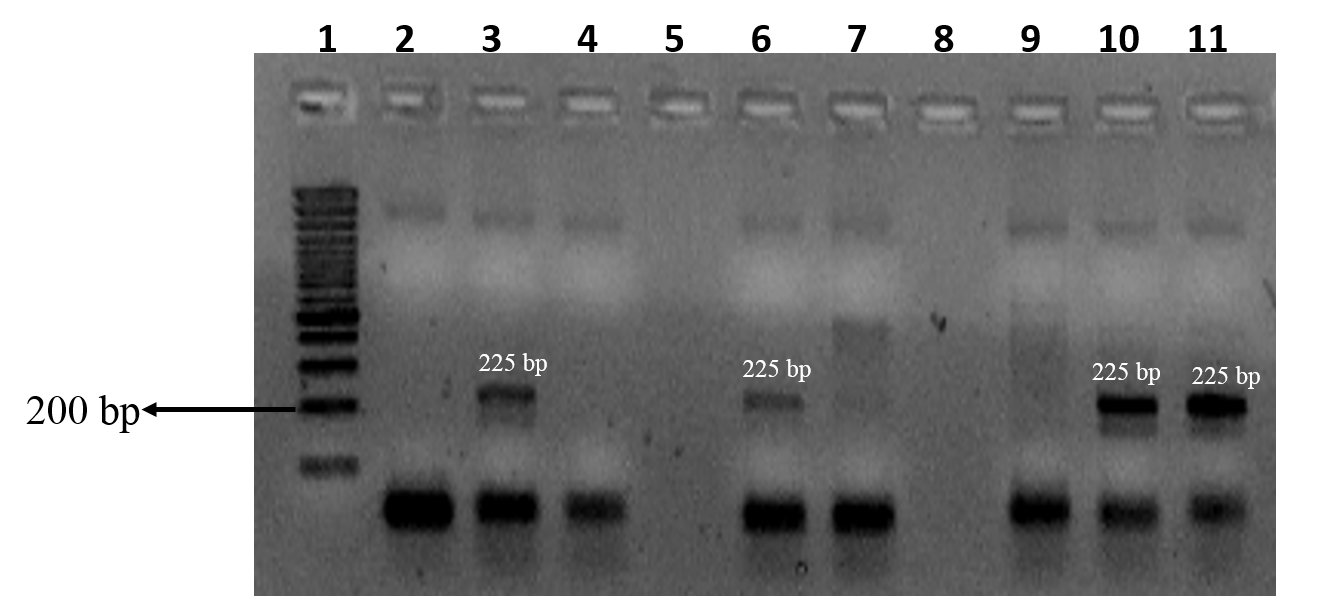

Supplement: S1 Fig — Lane 1: 100 bp ladder; 2: reaction without a template; 3: S. arvensis dicamba-resistant; 4: B. napus; 6, 7, 9, 10, 11 are hybrids generated via embryo rescue: 6, 10, 11 represent dicamba-tolerant hybrids, whereas, 7, 9 are dicamba-susceptible; 5 and 8: empty wells. (TIF) [file pone.0141418.s002.tif]

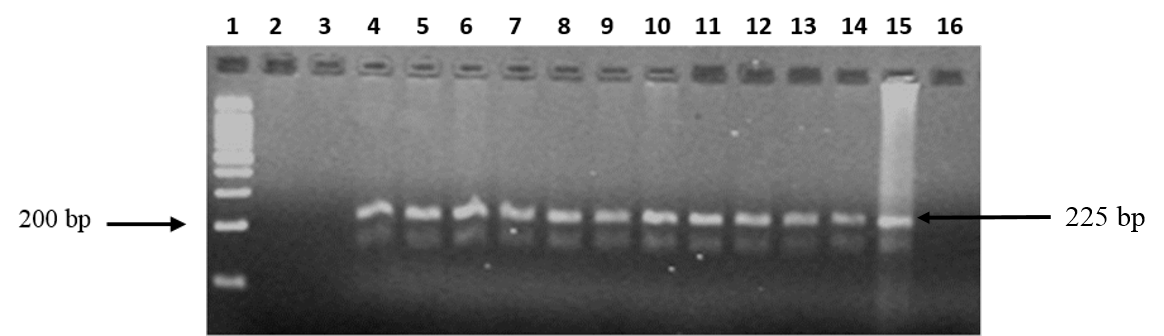

Supplement: S2 Fig — Lane 1: 100bp ladder; Lane 2: S. arvensis- dicamba susceptible; 3: wild-type B. napus; 4–13: Ten BC7F3 plants; 14, 15: S. arvensis-dicamba resistant; 16: reaction without a template. (TIF) [file pone.0141418.s003.tif]
